# Identification of missense and synonymous variants in Iranian patients suffering from autosomal dominant polycystic kidney disease

**DOI:** 10.1186/s12882-020-02069-0

**Published:** 2020-09-21

**Authors:** Fatemeh Khadangi, Adam Torkamanzehi, Mohammad Amin Kerachian

**Affiliations:** 1 Department of Biology, University of Sistan and Baluchestan, Zahedan, Iran; 2 Medical Genetics Research Center, Mashhad University of Medical Sciences, Mashhad, Iran; 3 Department of Medical Genetics, Faculty of Medicine, Mashhad University of Medical Sciences, Mashhad, Iran

**Keywords:** Autosomal dominant polycystic kidney disease, PKD1, Mutational analysis, Iranian

## Abstract

**Background:**

Autosomal dominant polycystic kidney disease (ADPKD), the predominant type of inherited kidney disorder, occurs due to *PKD1* and *PKD2* gene mutations. ADPKD diagnosis is made primarily by kidney imaging. However, molecular genetic analysis is required to confirm the diagnosis. It is critical to perform a molecular genetic analysis when the imaging diagnosis is uncertain, particularly in simplex cases (i.e. a single occurrence in a family), in people with remarkably mild symptoms, or in individuals with atypical presentations. The main aim of this study is to determine the frequency of *PKD1* gene mutations in Iranian patients with ADPKD diagnosis.

**Methods:**

Genomic DNA was extracted from blood samples from 22 ADPKD patients, who were referred to the Qaem Hospital in Mashhad, Iran. By using appropriate primers, 16 end exons of *PKD1* gene that are regional hotspots, were replicated with PCR. Then, PCR products were subjected to DNA directional Sanger sequencing.

**Results:**

The DNA sequencing in the patients has shown that exons 35, 36 and 37 were non- polymorphic, and that most mutations had occurred in exons 44 and 45. In two patients, an exon-intron boundary mutation had occurred in intron 44. Most of the variants were missense and synonymous types.

**Conclusion:**

In the present study, we have shown the occurrence of nine novel missense or synonymous variants in *PKD1* gene. These data could contribute to an improved diagnostic and genetic counseling in clinical settings.

## Background

One of the most prevalent inherited kidney disorders that affects both kidneys is autosomal dominant polycystic kidney disease (ADPKD), which leads to a progressive loss of kidney function and kidney failure [1]. About one to two infants in 1000 live at birth, and approximately 10% of people who undergo dialysis are affected by this disease [2, 3]. ADPKD occurs in two types including type I and type II, caused by *PKD1* and *PKD2* mutations, respectively [4, 5].

*PKD2* mutation causes end-stage renal disease at an average age of 74 years, which occurs in 10–15% of cases; on the other hand, *PKD1* mutation results in end-stage renal disease at an average age of 54 years which occurs in 80–90% of total cases of ADPKD. The latter is the more severe form of the disease [1, 3, 5]. Patients having end-stage kidney disease should receive renal replacement therapy (RRT) or dialysis to stay alive. However, dialysis has some limitations, including lack of vascular access, risks of vascular thrombosis, infections, diminished quality of life, and loss of the kidney biosynthetic functions [6]. Patients who were diagnosed with ADPKD before age of 30 and patients who have hypertension or hematuria before age of 35, have a worse renal outcome [7]. ADPKD diagnosis is typically carried out by kidney ultrasound imaging, computed tomography scan or magnetic resonance imaging; however, considering the similarity of ADPKD to other cystic kidney disorders, conventional imaging methods do not often lead to a definite diagnosis [1, 2]. Additionaly, molecular methods have an important role to confirm ADPKD diagnosis, especially in young kidney donors, patients with negative family history, individuals who present ADPKD with unusual symptoms in childhood and patients who have relatives suffering from this disorder [8, 9].

ADPKD is the most frequent genetic kidney disorder (frequency of about 0.1%), which results in 5–8% of end-stage renal diseases (ESRDs). ESRD is a progressive, disease with enlarged polycystic kidneys typically occuring in the late middle age [5]. Polycystin-1, is a large multidomain protein encoded by *PKD1* gene. It has domains and regions that are homologous with a number of different proteins [10]. Polycystin- 1 has been proposed to act as a G protein–coupled receptor [11]. Instead, polycystin-2 (the protein coded by PKD2) is homologous to an ion-channel subunit [12, 13]. Most cases of ADPKD leading to ESRD are caused by *PKD1* mutations [14]. Nevertheless, the genetic determination of the locus mutation has advanced slowly, due to the fact that *PKD1* contains a 12,906-bp coding sequence divided into 46 exons and that the 5′ region of the gene, from upstream of exon 1 to exon 33, is inserted in a complex genomic area and repeated more than 4 times on the same chromosome [15]. The polycystic kidney disease 1 gene encodes a 14 kb transcript and lies within a duplicated region on chromosome 16. Homologous sequences searches in a number of databases have found one partial cDNA and two genomic sequences with significant homology to both polycystin-1 and -2 [16].

The *PKD1*-like homologous gene (HG) has revealed a number of specific deletions and a low level of substitutions (about 2%) in comparison with *PKD1* [17]. The HG locus analysis of *PKD1* has been highly difficult. Thus, the quantity of identified *PKD1* mutations is still incomplete, with 82 modifications described in the Online Human Gene Mutation Database (HGMD) [18]. A multiple number of methods have been used to screen the repeated region [19–23], however, the 3′ area has received insufficient attention, with 57.3% of all mutations found in the single-copy area covering 20% of the coding region. *PKD2* (a less-complex gene) has revealed 41 mutations with potential effects of truncating and possibly inactivating the translated protein [24]. A discrete number of missense changes have also been described [19, 23–26]. Since numerous somatic mutations and a significant rate of formation of novel germline mutations are needed to explain cystogenesis [19], it has been proposed that infrequent mechanisms promote a high rate of *PKD1* mutations. A long polypyrimidine region in IVS21, which could theoretically form triplex DNA structures [27, 28], has been considered as a possible cause of mutations in downstream exonsequences [22]. These multiple substitutions and other modifications were described to match HG sequences, possibly indicating a gene conversion with the remotely located HG loci [21, 29]. *PKD1* gene (OMIM 601313) is located in the 16p13.3 chromosome region and consists of 46 exons. Exons 1–33 of *PKD1* replicates around 6 times in HG, which has challenged *PKD1* genetic analysis. Until January 2015, approximately 2322 *PKD1* sequence variants and 278 *PKD2* sequence variants were reported in ADPKD mutation databases, as well as 1177 and 211 human mutations in *PKD1* and *PKD2* sequences, respectively [16, 17]. Although mutation data for *PKD* genes of different populations are available, there are few reports for *PKD* mutations in the Iranian population. The main goal of this study was to establish the frequency of mutations in the *PKD1* gene obtained by PCR (Polymerase Chain Reaction) and DNA Sanger sequencing [30] in the Iranian patients with ADPKD diagnosis.

## Methods

### Patient selection

Twenty-two ADPKD patients were obtained from the Ghaem Hospital; (Mashhad, Iran) between April 2012 to March 2013. They were included after diagnosis and disease characteristics as ADPKD. The study was approved by ethics committee of Mashhad University of Medical Sciences. Before the blood sample were collected, all patients provided their informed consents.

We excluded patients later clinically diagnosed by Von Hippel-Lindau disease and Tuberous Sclerosis. In addition, patients without symptoms of polycystic kidney disease or those who had other syndromes were also excluded in this study.

### Amplification assay

Genomic DNA was extracted from 22 whole-blood samples using the standard salting-out method and it was quantified by NanoDrop 1000 (Thermo Fisher Scientific, Waltham, MA, USA). Eight-specific primers within the region of the exon 31–46 were designed with the Primer 3 software (Table 1). Sequences were checked for self- or inter-molecular annealing with a nucleic-acid-folding software (OligoAnalyzer 3.1). We performed local-alignment analyses with the BLAST program to confirm the specificity of the designed primers (http://www-ncbi-nlm-nih-gov.acces.bibl.ulaval.ca/tools/primer-blast). Bidirectional sequence analysis was conducted for all PCR amplicons.

**Table 1:** Coverage of the primers

Primers	Exons and introns
*PKD1*ex31–34	Int30-exo31-int31-exo32-int32-exo33-int33-exo34-int34
*PKD1*ex35–37	Int 34-exo35-int35-exo36-int36-exo37-int37
*PKD1*ex38–39	Int37-exo38-int38-exo39-int39
*PKD1*ex40–41	Int39-exo40-int40-exo41-int41
*PKD1*ex41–43	Int40-exo41-int41-exo42-int42-exo43
*PKD1*ex44–45	Int43-exo44-int44-exo45-int45
*PKD1*ex45–46	Int44-exo45-int45-exo46-int46

Amplification was performed in a thermal cycler, GeneAmp PCR System 9700 (Applied Biosystems, Massachusetts, USA), including 150 ng of genomic DNA, 10X PCR buffer, 2 mM MgCl2, 1 Unit Taq DNA polymerase (Genet Bio, South Korea), 0.2 mM dNTP mix, and 5 pmol of each primer in a final volume of 20 μl. Cycling parameters were as follows: an initial denaturation at 95 °C for 5 min, 35 cycles at 95 °C for 30 s, annealing for 30 s at 52 °C, 57 °C, 69 °C, 67 °C, 54 °C, 61 °C and 62 °C for primer#1 to #8 respectively, and a final extension step at 72 °C for 35 s, ended by a last extension at 72 °C for 5 min.

PCR products were analyzed by electrophoresis in a 1.5% agarose gel stained with ethidium bromide followed by Sanger sequencing reactions.

### Sanger sequencing

Sequencing products were run on an ABI 3130XL Genetic Analyzer (Macrogene Company South Korea), according to the manufacturer’s guidelines. Data analysis was performed with Chromas software version 2.6.5 (Technelysium, South Brisbane, Australia).

## Results

**Fig. 1:**
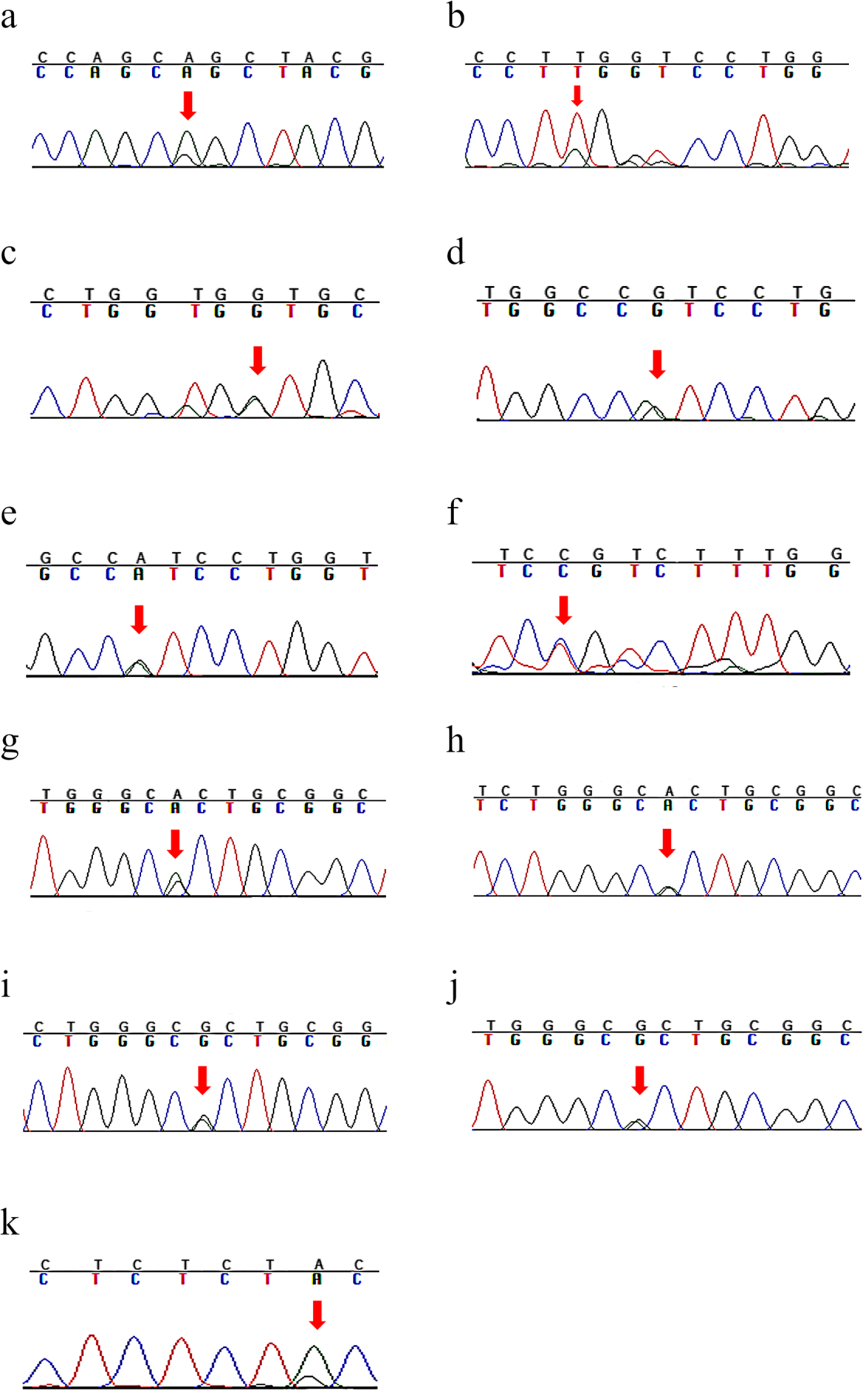
DNA sequencing results of Iranian patients with ADPKD. Patients including a) patient# 421 (p.Gln4005Arg); b) patient# 48.1 (p.Leu4031X); c) patient# 418.2 (p.Val4035Met); d) patient# 45.1 (p.Ile4045Val); e) patient# 410.2 (p.Ile4045Val); f) patient# 47.1 (p.Ser4013=); g) patient# 45.3 (p.Ala4092=); h) patient# 417.1 (p.Ala4092=); i) patient# 419.1(p.Ala4092=); j) patient# 410.1 (p.Ala4092=); k) patient# 422 (p.Thr4073Ala)

Twenty-two patients with an average age of 36.6 ± 7.3 years, suffering from ADPKD were studied. The sequencing results of the patientsare reported in Table 2 and Fig. 1. In patient 45.1, variations in rs10960 polymorphism in exon 44 led to the conversion of isoleucine to valine (p.Ile4045Val). This type of variation, considered as missense, was recorded in the PKDB database with a minor allele frequency of 0.239. Moreover, the single nucleotide variant (p.Ile4045Val), was also found in patient 410.2. In four patients, including 45.3, 410.1, 417.1, and 419.1, exon 45 had a synonymous mutation (p.Ala4092=) and was reported as rs3087632 with MAF: 0.262 in the database PKDB. Moreover, the missense mutation converting glutamine to arginine (p.Gln4005Arg) had occurred in exon 44 of the patient 421 and was recorded as uncertain significance in the PKDB database.

**Table 2:** Mutations and polymorphisms of PKD1 identified in this study

Patient ID	Region	cDNA Change	Amino Acid Change	Type	Clinical Significance
421	EX44	c.12014A > G	p.Gln4005Arg	Missense	Uncertain Significance
48.1	Ex44	c.12092 T > A	p.Leu4031X	Stop codon	​ Definitely Pathogenic
418.2	Ex44	c.12103G > A	p.Val4035Met	Missense	Possibly Damaging
45.1	EX44	c.12133A > G	p.Ile4045Val	Missense	Likely Neutral
410.2	EX44	c.12133A > G	p.Ile4045Val	Missense	Likely Neutral
47.1	Ex44	c.12039C > T	p.Ser4013=	Synonymous	Not Reported
45.3	EX45	c.12276A > G	p.Ala4092=	Synonymous	Likely Neutral
417.1	EX45	c.12276A > G	p.Ala4092=	Synonymous	Likely Neutral
419.1	EX45	c.12276A > G	p.Ala4092=	Synonymous	Likely Neutral
410.1	EX45	c.12276A > G	p.Ala4092=	Synonymous	Likely Neutral
422	EX45	12217A > G	p.Thr4073Ala	Missense	Likely Neutral

### Novel variants

The first variant was observed in patient 45.5. This variant caused a synonymous variant in exon 44 (p.Gly4068=). Patient 47.1, a variant of rs200796474 was also synonymous, with a serine converted to serine (p.Ser4013=). A leucine to stop codon mutation, was observed in exon 44 of patient 48.1 (p.Leu4031X). In patient 411.2, a missense converting arginine to leucine was observed in exon 45 and the missense mutation converting arginine to leucine was also found in the same exon of the same patient (CGT/CTT). The missense change converting valine to methionine occurred in exon 44 of patient 418.2 (p.Val4035Met). In addition, the missense variation converting threonine to alanine was found in some part of the exon 45 in the patient 422 (p.Thr4073Ala)(Table 2).

### In silico functional analysis

Nucleotide changes in the *PKD1* gene was determined based on reference genomic sequemces NC_000016.10. The detected sequence variations reported in this study were checked with the list of Autosomal Dominant Polycystic Kidney Disease Mutation Database (PKDB) and *PKD* gene variants in the Human Gene Mutation Database (HGMD) [31].

The pathogenicity prediction of novel variations were analyzed by Mutation Taster [32]. We checked related protein products for sequence and length alteration by altered CDS (NM_001009944) using expasy translate tools. The prediction obtained of the potential effect of each variant has been shown in Table 2. In the current study, mutations were named based on CDS according to standard mutation nomenclature for molecular diagnostic aims.

The UniProt database, UniProtKB ID Q8IYM9 (http://www.uniprot.org), the NCBI dbSNP database (https://www.ncbi.nlm.nih.gov/SNP/), and 1000 Genomes (http://www.1000genomes.org/) have been used to retrieve polymorphism data. Functional effects of SNPs were predicted using Polyphen-2 (http://genetics.bwh.harvard.edu/pp2).

## Discussion

To date, 2322 pathogenic meuations for *PKD1* and 278 for *PKD2* have been reported in the PKDB [33] but their relative frequencies are unknown. Moreover, Daoust et al., identified a family in the French-Canadian population in which a classical clinical presentation of ADPKD resulted from a mutation at a locus genetically distinct from all the previously described loci for this disease. This suggests an existence of a third genetic locus for ADPKD [5].

In the current study, 16 end exons of *PKD1* gene were studied. The sequencing results have shown that exons 35, 36 and 36 were non-polymorphic, with no mutations, and the most mutations occurred in exons 44 and 45. In most of the patients, variants were mostly missense and same-sense types. Our results have shown that there is no definite hot spot in *PKD1* and thus, a complete *PKD1* mutation analysis is needed for genetic diagnosis of ADPKD in the Iranian patients. Our newly detected mutations in the Iranian population have made the *PKD* mutation database richer, a result of great importance in the genetic consultation of ADPKD patients.

Regarding the large genes involved in ADPKD, screening all of their regions would be expensive and time-consuming; hence, to overcome this issue a database could be generated for mutations of polycystic kidney disease among the Iranian population to determine the most common mutations and to characterize mutation hot spots in this population. Furthermore, considering the clinical similarity of ADPKD with other kidney cystic diseases, causing incorrect clinical diagnosis in the absence of familial history, molecular study for *PKD1* with or without *PKD2* in suspected patients is recommended.

Identified pathogenic mutations in the present study could be confirmed in future studies with more ADPKD families. Besides, genotype-phenotype correlation studies could be performed to determine the severity of each variant and the outcome of patients associated with a specific variant.

## Conclusion

In the current study, we demonstrated nine novel missense or synonymous variants in *PKD1*. These data will contribute to an improved diagnostic and genetic counseling in clinical settings.

## Data Availability

The datasets generated and/or analysed during the current study are available in the GenBank repository, [BankIt2382235 BSeq#1 MT993938]. Any additional information related to this study is available from the author for correspondence upon reasonable request.

## Abbreviations

ADPKD: Autosomal dominant polycystic kidney disease
ESRDs: End-stage renal diseases
PCR: Polymerase chain reaction
PKDB: Polycystic Kidney Disease Mutation Database
HGMD: Human Gene Mutation Database
